# DEIM-SFA: A Multi-Module Enhanced Model for Accurate Detection of Weld Surface Defects

**DOI:** 10.3390/s25206314

**Published:** 2025-10-13

**Authors:** Yan Sun, Yingjie Xie, Ran Peng, Yifan Zhang, Wei Chen, Yan Guo

**Affiliations:** 1 College of Information Engineering, Sichuan Agricultural University, Ya’an 625000, China; 202307489@stu.sicau.edu.cn (Y.S.); 202205778@stu.sicau.edu.cn (Y.X.); pengran@stu.sicau.edu.cn (R.P.); marcusky@stu.sicau.edu.cn (Y.Z.); chenwei1@stu.sicau.edu.cn (W.C.); 2 Ya’an Agricultural Engineering Technology Research Center, Ya’an 625000, China

**Keywords:** DEIM, feature fusion, industrial vision, welding defect detection

## Abstract

High-precision automated detection of metal welding defects is critical to ensuring structural safety and reliability in modern manufacturing. However, existing methods often struggle with insufficient fine-grained feature retention, low efficiency in multi-scale information fusion, and vulnerability to complex background interference, resulting in low detection accuracy. This work addresses the limitations by introducing the DEIM-SFA, a novel detection framework designed for automated visual inspection in industrial machine vision sensors. The model introduces a novel structure-aware dynamic convolution (SPD-Conv), effectively focusing on the fine-grained structure of defects while suppressing background noise interference; an innovative multi-scale dynamic fusion pyramid (FTPN) architecture is designed to achieve efficient and adaptive aggregation of feature information from different receptive fields, ensuring consistent detection of multi-scale targets; combined with a lightweight and efficient multi-scale attention module (EMA), this further enhances the model’s ability to locate salient regions in complex scenarios. The network is evaluated on a self-built welding defect dataset. Experimental results show that DEIM-SFA achieves a 3.9% improvement in mAP50 compared to the baseline model, mAP75 by 4.3%, mAP50–95 by 3.7%, and Recall by 1.4%. The model exhibits consistently significant superiority in detection accuracy across targets of various sizes, while maintaining well-balanced model complexity and inference efficiency, comprehensively surpassing existing state-of-the-art (SOTA) methods.

## 1. Introduction

Metal welding, as a critical process in modern manufacturing, is widely applied in industries such as construction and rail transport [1]. The quality of welding directly impacts the structural strength, service life, and operational safety of products, thereby determining the reliability of manufacturing systems and the overall performance of engineering projects [2]. In the context of increasingly complex industrial structures and continuously rising performance requirements, high-quality welding processes are not only a crucial foundation for ensuring the stable operation of equipment but also a key enabler for advancing high-end manufacturing, efficient assembly, and sustainable development. However, due to the involvement of multiple complex factors in the welding process, including material properties, welding process parameters, and external environmental conditions, welding defects are complex to avoid altogether [3]. These defects not only weaken the mechanical properties of the welded joint but may also lead to equipment failure or even safety accidents, particularly in applications with extremely high structural integrity requirements, such as pressure vessels and rail transit. Even minor welding defects can result in catastrophic consequences. Therefore, welding defect detection is a critical step in improving welding process productivity and quality [4]. Common defect types, such as cracks, porosity, and spatter [5], are often difficult to detect in the early stages but can easily become stress concentration points during service, leading to fatigue failure, stress corrosion cracking, or even complete system dysfunction. Additionally, such hidden defects significantly increase post-production maintenance and operational costs, severely limiting quality control and sustainable development in manufacturing. Traditional welding defect detection primarily relies on non-destructive testing technologies such as X-rays, ultrasonic waves, thermographic testing, and visual inspection [6]. Although these techniques provide a certain level of accuracy, performance is limited by the complexity of defects and irregular surface textures, leading to challenges in accuracy, real-time performance, and cost control. Furthermore, these methods rely heavily on manual operators, which in large-scale or high-precision inspection scenarios often leads to reduced detection efficiency, increased labour costs, and limited data processing capabilities [7], particularly in complex structures or large-scale weld inspections, where modern manufacturing demands are not met. While some high-precision detection methods can identify minute defects, the application of such methods is often associated with high costs and low detection efficiency. Conversely, low-cost detection solutions may struggle to meet high-precision detection requirements. Therefore, achieving a reasonable trade-off between accuracy, processing speed, and resource cost is still a core challenge for welding defect detection technology.

With the rapid development of artificial intelligence technology, defect detection based on deep learning has gradually become the core content of machine detection. Deep learning object detection algorithms utilise convolutional neural networks (CNNs) to convert raw input information into more abstract, higher-dimensional features for learning, with strong feature representation and high-dimensional feature generalization capabilities that enable adaptation to more complex scenarios [8]. The application of deep learning methods to welding defect detection not only enables automatic identification and classification of defect types but also significantly improves detection efficiency and accuracy while reducing errors caused by human intervention, providing a revolutionary solution for welding defect detection [9]. These techniques can currently be roughly divided into two categories: the two-stage object detection algorithm, whose detection process is divided into two steps—candidate region generation and subsequent classification—with typical representatives including Fast-RCNN and Mask-RCNN [10,11]; its main feature is that it generates a set of candidate regions in the first stage, followed by classification and bounding box regression of these candidate regions in the second stage. These methods typically achieve high detection accuracy and are particularly suitable for tasks requiring high detection accuracy. However, slow inference speeds limit their applicability to real-time detection tasks [12]. In contrast, one-stage detection methods represented by the You Only Look Once (YOLO) series [13,14,15,16,17], Real-Time Detection Transformer (RT-DETR) [18], and Single-Shot MultiBox Detector (SSD) [19] adopt an end-to-end architecture, directly predicting both the object category and location in the image without an intermediate candidate region generation process. These methods significantly improve inference speed and are suitable for applications with high real-time requirements. In recent years, such methods have garnered widespread attention for balancing detection accuracy and efficiency. However, these general-purpose object detection models still face significant challenges when directly applied to the highly specialized, fine-grained task of identifying welding defects. Welding defects typically exhibit petite sizes, blurred boundaries, irregular shapes, and textures highly similar to the weld seam background [20].

In recent years, visual Transformer-based models have achieved impressive accuracy in object detection tasks [21]. As the latest proposed next-generation end-to-end detection framework, DEIM (DETR with Improved Matching) demonstrates excellent detection performance in welding defect detection tasks. By introducing an optimized matching strategy, it significantly improves training convergence speed and matching stability, addressing the shortcomings of the original DETR in target allocation [22]. Additionally, DEIM designs a matching-aware loss function that dynamically adjusts the weights of low-quality matching samples. Consequently, the model’s resilience in complicated environments is improved, leveraging the Transformer’s global modelling capabilities, DEIM can better capture defect regions with complex structures and substantial background interference in weld seam images. However, despite DEIM’s advantages in detection accuracy and end-to-end simplified workflow, its performance in weld defect detection still faces certain challenges. The standard convolutional backbone network adopted by DEIM has limited perception capabilities for minor, low-contrast defects, making the complete extraction of fine-grained semantic information challenging. Additionally, its feature fusion strategy is relatively shallow, posing a risk of missed detections for multi-scale defects, especially those of petite sizes [23]. Furthermore, while the global attention mechanism of the standard Transformer excels in overall context modelling, its perception capabilities remain insufficient when handling fine-grained defects such as minor cracks on weld surfaces, particularly in complex scenarios where defects have low contrast with the background, leading to feature expression confusion. In addition, the training process demonstrates slow convergence, requiring numerous iterations to achieve stability and thereby increasing both training time and computational cost. The one-to-one matching strategy used by the Hungarian algorithm results in a minimal number of positive samples and low matching quality, especially in the field of small object detection, limiting the model’s learning effectiveness [24,25].

In order to solve the aforementioned problems, this study offers a multi-module collaborative network for metal welding flaw detection, DEIM-SFA, which integrates an improved DEIM algorithm. First, to augment the feature retention and small object identification capabilities of the primary network during the downsampling stage, a spatial-depth conversion convolution (SPD-Conv) module is introduced at certain downsampling positions in the HGNetv2 main network. Through a sub-pixel reordering mechanism [26], spatial information is efficiently mapped to the channel dimension, thereby realizing lossless spatial-to-channel rearrangement and enabling efficient information transmission. Second, to strengthen feature learning and mitigate overfitting, the neck incorporates an FTPN module designed with a multi-level feature fusion strategy. This structure serves as the encoder component of the DEIM model, using deformable convolutional kernels to adapt to objects of different scales and effectively process features of varying scales. Finally, to more effectively retain channel information and enhance spatial modelling capabilities, an efficient attention mechanism is introduced, EMA (Efficient Multi-Scale Attention) [27], which rearranges features and maps some channels to the batch dimension, enabling spatial semantic information to be expressed more evenly across each feature group, thereby enhancing the model’s contextual awareness. According to experimental results, the model enhances information retention, multi-scale feature fusion, and noise suppression in images, making the sensing process more robust to complex backgrounds and environmental disturbances. The following are our primary contributions:

1. Proposing a downsampling method with a spatial-information-preserving design. The network backbone was designed to integrate the SPD-Conv module. Unlike traditional downsampling operations, SPD-Conv can rearrange feature maps without losing information, retaining fine spatial details and encoding them into the channel dimension, thereby providing the subsequent network with richer feature representations that are conducive to the identification of minor defects.
2. Designing a multi-scale dynamic feature fusion neck: The FTPN network was constructed as the model neck to replace the original feature fusion module. FTPN leverages its unique multi-scale dynamic focusing and feature diffusion mechanisms to adaptively aggregate and enhance features from different layers, significantly improving the model’s ability to model defects of varying sizes and blurred edges.
3. Incorporating an efficient context-aware attention mechanism: The EMA module is added to the network. This mechanism enables the model to efficiently establish global context dependencies through channel reorganisation and grouping across spatial dimensions, thereby more accurately focusing on the actual defect regions and effectively suppressing interference from complex background textures.
4. Constructing a high-quality industrial welding defect dataset: To realistically evaluate model performance and promote research in this field, an industrial welding defect dataset comprising 2733 images across six categories was collected, organised, and annotated. This dataset covers a wide range of welding defects encountered in actual applications and can serve as a rigorous standard for further research and verification.

## 2. Methodology

The infrastructure of the DEIM-SFA network is based on the current state-of-the-art single-stage object detector, DEIM (DETR with Improved Matching) [25]. DEIM was selected as the benchmark model due to its excellent balance between detection accuracy and inference speed. The model adopts a highly modular design, offering five pre-configured versions: nano (n), small (s), medium (m), large (l), and extra-large (x). After comparative analysis, the DEIMs version was chosen as the fundamental framework for this investigation, striking the best possible compromise between model complexity and detection performance. The DEIM architecture consists of three main parts: a detection head with sophisticated optimization techniques, a bidirectional fusion neck, and an effective backbone network. The backbone network is HGNetv2, composed of a Stem module, multiple HG-Blocks, and several convolutional modules, which are used to achieve multi-scale hierarchical feature extraction. These multi-scale features are then fed into a neck that combines a Feature Pyramid Network (FPN) and a Path Aggregation Network (PANet) [28,29], which deeply fuses high-level semantic information with low-level spatial details through bidirectional information flow to generate feature maps rich in contextual information. Finally, the detection head uses these fused features for bounding box regression and class prediction. It combines two key training strategies for performance optimisation: the Dense One-to-One Matching strategy optimises the traditional Hungarian matching [30], assigning each object an appropriate prediction head to avoid neglecting ‘small objects’ or ‘minority class objects’; a loss function sensitive to matching quality is introduced, enabling dynamic adjustment of weights for suboptimal samples to further enhance the model’s detection robustness in complex backgrounds.

### 2.1. Improved Overall Network Architecture

**Figure 1:**
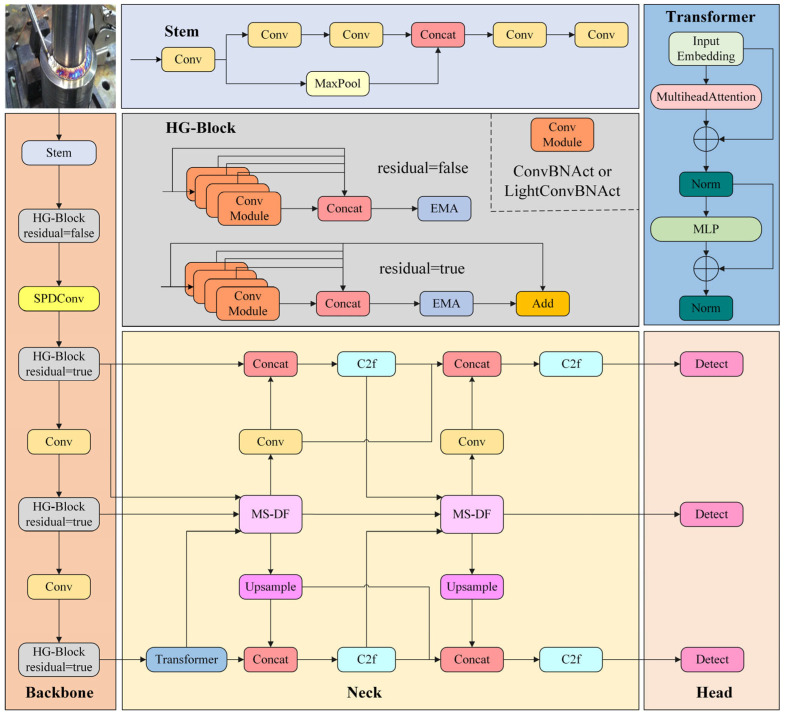
Overall architecture of DEIM-SFA.

The overall structure of DEIM-SFA is shown in Figure 1. To enhance the network’s performance in detecting targets of different scales, this paper modifies the HGNetv2 backbone structure of the original DEIM model. First, in the backbone network, the original Stage 2 spatial downsampling module is replaced with an SPD-Conv module. SPD-Conv achieves more efficient information compression through spatial pyramid depth convolution while preserving rich spatial details and multi-scale semantic perception capabilities. Since SPD-Conv requires a large receptive field to capture spatial pyramid features, its effects are not prominent in shallow feature maps. Therefore, it is only replaced in Stage 2 to achieve the best balance. Secondly, to address the issue of target details being weakened during continuous convolution, the EMA attention mechanism is introduced into all HG-Block modules. This module effectively improves the network’s modeling capacity of small targets and conspicuous features in complex background by adaptively increasing spatial and channel information and directing the network to concentrate on crucial locations. Finally, to further improve feature fusion capabilities, we replace the neck structure based on FPN and PANet in the original model with an innovatively improved FTPN architecture. The FTPN structure combines multi-scale diffusion (MS-DF) with a Transformer encoder, which enables precise fusion of features across scales while incorporating global context, enhancing the recognition of defects of varying sizes and types. The selective application of the Transformer encoder to deep features ensures a balance between computational efficiency and performance. Collectively, this integration of three modules provides a unique framework that addresses the specific challenges of welding defect detection—including small target sizes, complex backgrounds, and diverse defect types—and provides superior accuracy and robustness compared to conventional approaches.

Our framework is the first to systematically integrate SPD-Conv, FTPN, and EMA within the DEIM network. This integration is theoretically motivated by the complementary strengths of the modules: SPD-Conv enhances local detail preservation, FTPN strengthens multi-scale fusion, and EMA improves contextual attention. Their joint design enables a balanced architecture tailored for welding defect detection. The model achieves a good balance between accuracy and computational cost, requiring only 11.87 million parameters and 36.35 GFLOPs. This lightweight design shows potential for deployment on edge devices and embedded sensors, which may enable real-time online monitoring in industrial environments.

### 2.2. SPD-Conv Module for Fine-Grained Feature Retention

In the task of detecting defects in metal welding, the accurate identification of small defects (such as welding cracks and spatter) has always been a significant challenge. Compared to large-scale structural changes, some defects often exhibit characteristics such as small size, irregular shape, and blurred boundaries [31], resulting in them covering merely a small portion of the pixels in the picture. This leads to insufficient effective feature representation, thereby affecting the model’s detection accuracy. To solve these concerns, the SPD-Conv module was deployed at the downsampling stage of the backbone network, replacing the original stride-based convolution and pooling layers. The theoretical motivation for this choice lies in its ability to preserve fine-grained spatial details while achieving efficient multi-scale feature representation. The general idea of SPD-Conv is that a Space-to-Depth transformation is first applied to redistribute spatial information into the channel dimension, and a stride-free convolution is then used to compress and fuse these features, thereby reducing spatial resolution while avoiding the loss of small-target details. Unlike traditional fixed convolutions, SPD-Conv dynamically adjusts its receptive field based on the geometric properties of different regions within the input image, effectively improving the modelling accuracy of weld defect boundaries, shapes, and distribution characteristics. Additionally, SPD-Conv enhances the expression capability of shallow-layer features while maintaining network lightweightness, effectively improving small object detection performance and overall robustness in steel weld defect detection tasks.

**Figure 2:**
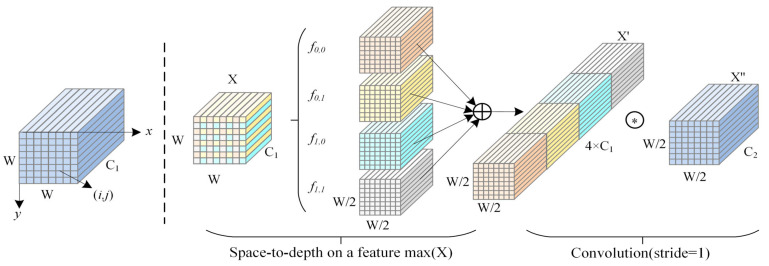
Structure of SPD-Conv when S = 2. The symbol ∗ denotes the convolution operation applied to X″ with a stride of 1.

The SPD-Conv module consists of two core components: the Space-to-Depth Transformation Layer and a volume-preserving layer [32]. These components enhance feature expression capabilities from both the spatial dimension and channel volume perspectives, thereby improving the model’s ability to model structural details. The architecture of the SPD-Conv module is shown in Figure 2. First, a Space-to-Depth transformation is applied to the input feature map by specifying a stride S, which partitions and rearranges the spatial dimensions accordingly. The input feature map X has a shape of W × W × C1; it is divided into S2 sub-feature maps *f*(x, y), whose range is as follows:

*f*_0,0_ = *X*[0:*W*:*S*, 0:*W*:*S*], …, *f_S_*_−1,0_ = *X*[*S* − 1:*W*:*S*, 0:*W*:*S*]
*f*_0,1_ = *X*[0:*W*:*S*, 1*:W*:*S*], …, *f_S_*_−1,1_ = *X*[*S* − 1:*W*:*S*, 1:*W*:*S*]
…
*f*_0,*S*−1_ = *X*[0:*W*:*S*, *S* − 1:*W*:*S*], …, *f_S_*_−1,*S*−1_
*= X*[*S* − 1:*W*:*S*, *S* − 1:*W*:*S*] (1)

These sub-feature maps are then concatenated along the channel axis to generate a feature map of size $\frac{w}{2}$ × $\frac{w}{2}$ × S^2^C_1_. When S = 2, the input is divided into four sub-feature maps *f*(0,0), *f*(0,1), *f*(1,0), and *f*(1,1), each of dimension $\frac{w}{2}$ × $\frac{w}{2}$ × C_1_. After concatenation, the resulting feature map has four times the original number of channels. This process effectively preserves the fine-grained structural information of the original space. It encodes it into the channel dimension in a more compact form, thereby significantly enhancing the network’s ability to model local textures and edge details.

Finally, a stride-free convolution layer with a D2 filter (stride = 1) [33] was added to X′ to aggregate and compress high-dimensional channel features, extracting discriminative semantic representations and transforming *X′* into *X″*, as shown in the following equation:

*X″* = *Conv* (*X′*, *filters* = *C*_2_, *stride* = 1) (2)

Unlike traditional downsampling using stride convolution, SPD-Conv avoids further compression of the spatial dimension. This convolution operation aims to retain as much distinctive feature information as possible, effectively preventing the loss of small target information. The final output feature map X has dimensions of $\frac{w}{2}$ × $\frac{w}{2}$ × C_2_.

### 2.3. FTPN Neck Network for Deep Multi-Scale Fusion

**Figure 3:**
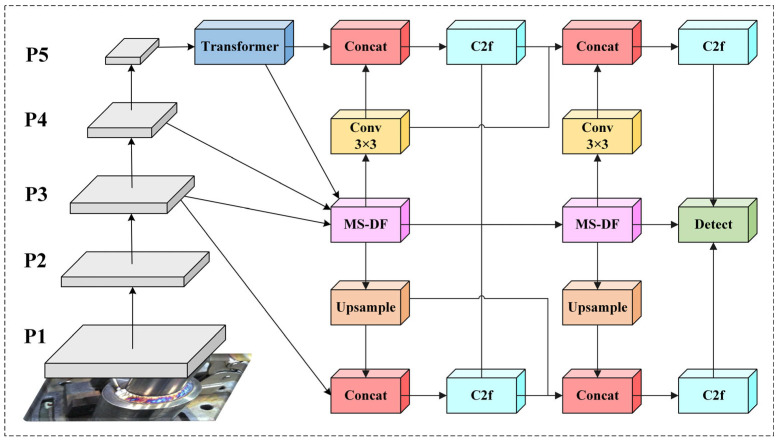
Structure of FTPN.

In welding defect detection, the choice of the Feature-focused Diffusion Pyramid Network (FTPN) as the neck architecture is theoretically motivated by the need to enhance multi-scale feature representation and cross-level interaction. Traditional neck networks based on FPN and PANet employ linear top-down and bottom-up pathways. While this design establishes cross-level connections, the depth and breadth of feature interactions are limited, leading to insufficient non-linear fusion of high-resolution details from shallow layers with high-order semantic information from deep layers, thereby limiting the model’s ability to characterise defects with extreme sizes or ambiguous morphologies [34,35]. To overcome this bottleneck, FTPN addresses this limitation by introducing an iterative, bidirectional feature refinement mechanism through the Multi-Scale Dynamic Fusion (MS-DF) module, enabling deeper and more effective multi-scale feature integration while minimizing semantic information loss. Furthermore, a lightweight Transformer encoder is applied to the highest-level feature map (P5), which contains the strongest semantic information, in order to enhance global context modeling and establish long-range dependencies via self-attention. This significantly improves the model’s ability to identify welding defects in complex backgrounds. Its structural diagram is shown in Figure 3. The Transformer encoder process can be expressed as:

*Y* = *Reshape* (*TransformerEncoder*(*Flatten*(*X*) + *PE*)) (3)

where *X* represents the input high-level feature map, *Flatten* () denotes flattening the input feature map along the spatial dimension into a sequence format, and PE is a learnable position encoding used to introduce spatial position information and preserve positional signals. *TransformerEncoder* () represents the main body of the encoder, typically consisting of multiple layers of multi-head self-attention and feedforward networks, used to model global dependencies within the sequence. *Reshape* () is used to restore the sequence features output by the encoder to their original spatial feature map shape; the final output Y is the high-level semantic feature map. Through this mechanism, the Transformer encoder significantly boosts the feature map’s spatial coherence and semantic representation capacity, providing more discriminative semantic support for subsequent multi-scale feature fusion and defect detection. Then, the three feature maps (P3, P4, P5) are fused at multiple scales through the first-stage MS-DF module, and the fused features are upsampled to the P3 layer and downsampled to the P5 layer for feature propagation. Subsequently, the second-stage MS-DF module performs a second multi-scale fusion on the output of the first stage and again conducts bidirectional feature propagation. Finally, three enhanced multi-scale feature maps are output, achieving efficient multi-scale feature fusion and bidirectional information flow.

**Figure 4:**
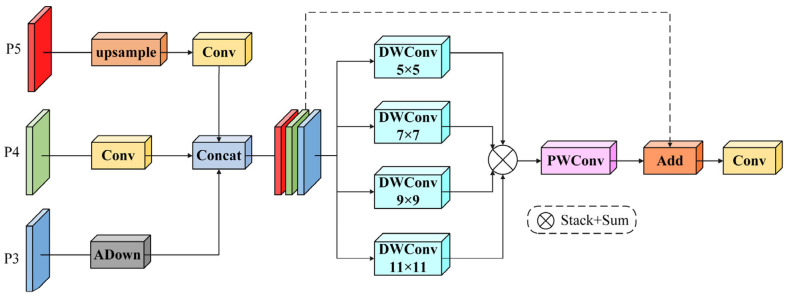
Structure of MS-DF.

In FTPN, the MS-DF module is its core innovative unit, specifically designed to process feature inputs at three different scales. The MS-DF dynamically integrates cross-scale features by simultaneously analyzing both adjacent-level and same-level feature representations, effectively integrating cross-layer information to compensate for the lack of spatial detail in shallow feature maps, enabling more accurate capture of defect changes. The structure is shown in Figure 4. This module first divides the input feature map and performs different operations based on scale differences. The input P3 layer undergoes feature downsampling via the Adown module, reducing the input feature size while extracting more representative features, thereby preserving low-level detail information. The input P4 layer passes through a convolutional layer, while the input P5 layer first undergoes upsampling followed by convolution. Subsequently, the concatenated features are processed through multi-core deep convolutional aggregation, which can be formally expressed as:

(4) $$Y\;=\;W_{1\times 1}\times (C+\sum_{k\in \left\{3,5,7,9\right\}}{DWConv}_{k}\left(C\right))$$

where *C* denotes the concatenated feature maps. Residual connections are used to enhance feature expression, and finally, a 1 × 1 convolution is applied to adjust the output dimensions, ensuring that low-level high-resolution features and high-level semantic features are complementary and enhance each other under an optimized fusion strategy. The design of the MS-DF module implicitly adjusts the contribution of features across layers. Through an optimized feature fusion strategy, the high-frequency details of shallow features and the abstract semantics of deep features are deeply integrated, significantly improving the model’s context modeling capabilities.

### 2.4. Efficient Multi-Scale Attention (EMA) Mechanism for Feature Refinement

**Figure 5:**
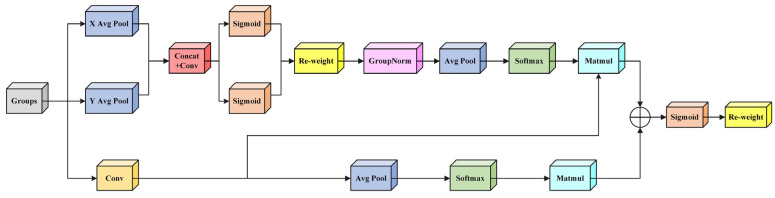
Structure of EMA.

The selection of the Efficient Multi-scale Attention (EMA) mechanism is theoretically motivated by the intrinsic characteristics of welding defect detection tasks. The baseline model employs the HGNetv2 backbone network, which integrates the classic Efficient Squeeze-and-Excitation (ESE) attention mechanism [36]. Its core mechanism utilizes global spatial pooling operations to achieve dimensional compression from two-dimensional features to one-dimensional channel descriptors, thereby establishing dependencies between channels. While this method effectively captures inter-channel relationships, it comes at the cost of losing spatial structural information. For metal welding defect detection tasks, defects such as cracks, porosity, and spatter exhibit highly irregular and localised spatial distributions. Suppose spatial position information is lost during feature extraction. In that case, the model will struggle to accurately identify the prominent features of these local regions, leading to reduced recognition capabilities for small targets or weakly textured defects. This is a suboptimal choice for dense prediction tasks that require precise spatial localization. By contrast, EMA is explicitly designed to address this shortcoming. Its theoretical advantage lies in its joint modeling of multi-scale spatial dependencies and cross-channel interactions. Through directional pooling along height and width, EMA preserves global spatial context while maintaining orientation-aware representations. At the same time, local convolutions capture fine-grained structural details, and the cross-spatial attention mechanism adaptively calibrates features across regions. This synergistic design allows EMA to retain spatial awareness without sacrificing computational efficiency. The network architecture is shown in Figure 5. The EMA module is composed of two parts: parallel subnetworks and cross-spatial attention mechanisms, achieving synergistic optimization of directional perception and regional modeling. The spatial awareness of EMA is realized through directional pooling and cross-spatial interaction. Specifically, the input features are grouped along the channel dimension, and three parallel paths are used to extract attention channel weights. In these three paths, the first two paths process features through 1 × 1 convolutions, employing two separate 1D adaptive pooling modules that aggregate features globally along each spatial dimension (height and width) for feature encoding, to capture global contextual information in different spatial directions. The third path employs 3 × 3 convolutions to capture local spatial features. For the path, 3 × 3 convolutions extract local detail features, and cross-spatial interaction paths (including feature calibration and matrix interaction) are used. Then, cross-spatial attention fusion is achieved through an innovative bidirectional softmax matrix multiplication, and finally, the original features are adaptively adjusted using dynamically generated attention weights. The entire structure reduces computational complexity through batch processing. By exploiting hierarchical feature extraction across multiple scales and synergistic spatial-channel interactions, the framework achieves efficient feature enhancement while maintaining the input and output dimensions unchanged.

The integration of EMA strengthens the network’s capacity to capture multi-scale contextual information and adaptively adjust channel feature responses. In the HGNetv2 backbone network, this improves the model’s ability to model long-range dependencies and multi-scale contextual information, significantly enhancing performance in object detection tasks. This module enables the network to focus on more discriminative features, thereby improving overall performance. Experimental results show that using EMA as the feature aggregation method achieves superior detection and segmentation accuracy compared to the original model’s ESE aggregation method in scenes with complex backgrounds. Overall, the EMA attention mechanism serves as an efficient feature aggregation module for the HGNetv2 backbone network, enhancing feature representation capabilities while maintaining computational efficiency, thereby providing a robust feature foundation for downstream visual tasks.

## 3. Experiments and Analysis

### 3.1. Dataset

**Figure 6:**
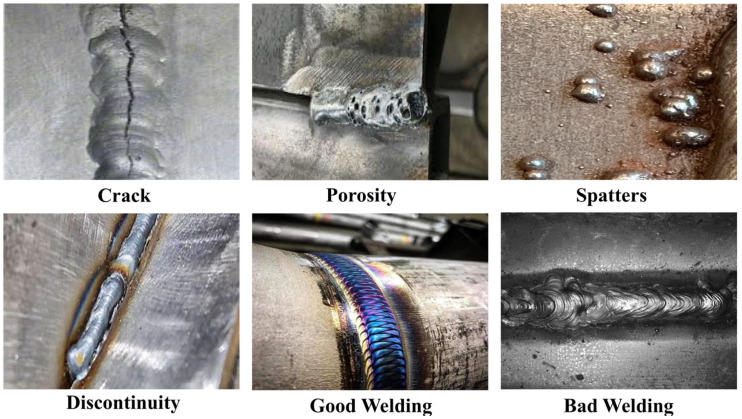
Six types of defects in the data set.

The scarcity and imbalance of welding defect datasets are common challenges. Many studies include underrepresented rare defect categories, such as pores or cracks. This class imbalance can distort model training, leading to biased predictions and reduced generalisation ability [19]. To verify the algorithm’s efficacy, a specific metal welding defect dataset is constructed. This dataset was built by integrating multiple relevant public datasets and consists of images annotated by professionals according to industry standards using LabelImg. The images were resized to a consistent resolution of 640 × 640 pixels to meet the input requirements of deep learning models. It contains a total of 2733 images, with hundreds of sample images in each category. The dataset is divided into training and validation sets in an 8:2 ratio. As shown in Figure 6, the dataset includes six key categories aimed at comprehensively simulating real industrial scenarios, namely Crack, Porosity, Spatter, Discontinuity, and two additional categories—Good Welding and Bad Welding—to assess the model’s ability to distinguish between qualified and unqualified welds.

During data collection, we specifically considered diverse industrial scenarios, with data sources covering different welding processes, material types, and lighting conditions, including indoor and outdoor environments, varying background noise levels, and complex texture conditions. These heterogeneous conditions effectively enhance the dataset’s diversity and real-world complexity, thus enhancing the model’s robustness and ability to generalize. As is common in welding inspection datasets, the self-built dataset also inherently suffers from class imbalance, since certain defects (bad welding, spatters) occur more frequently in practice, while others (porosity, cracks) are relatively rare. To mitigate this issue and prevent the model from being biased toward majority classes, targeted data augmentation techniques—including random rotation, flipping, cropping, and contrast adjustment—were primarily applied to minority classes. The training distribution was adjusted to approximately 10:7:6:5:4:4 (Bad Welding:Spatters:Good Welding:Discontinuity:Crack:Porosity). This strategy reduced the original imbalance to about 2.5:1, effectively alleviating the bias toward majority classes while preserving the natural differences in defect frequency commonly observed in industrial practice. Such a balanced-yet-realistic distribution enhances the reliability of model training and ensures better generalization to real-world welding inspection scenarios. This strategy not only balanced the class distribution, but also effectively expanded the dataset size, alleviating the risk of overfitting. Beyond balancing the class distribution, these augmentation techniques increase data diversity by simulating variations in real-world welding environments, thereby enhancing the model’s robustness and generalization ability. In addition, the detection accuracy of subtle defects is further improved, enabling the model to correctly identify instances that might otherwise be misclassified due to limited variability in the original dataset. To reduce computational complexity and satisfy the input requirements of deep learning models, all photos were reduced to a consistent dimension. Additionally, to eliminate colour biases caused by different capture devices and lighting conditions, channel histogram equalisation was applied to the input images. This technique enhances contrast by redistributing intensity levels within the image, particularly in areas with weak defect signals. Studies using histogram equalisation have shown that by more uniformly distributing pixel intensities, the detection effectiveness of defects such as cracks and porosity can be significantly improved [20]. Through these preprocessing steps, data quality is enhanced, enabling the model to keep stable weld defect detection capabilities across various metal types, welding procedures, and lighting conditions.

### 3.2. Experimental Environment Evaluation Metrics

After constructing the dataset, this study used the same hardware and software environment to ensure the fairness and comparability of the experimental results. The experimental environment included an NVIDIA RTX 3090 graphics processing unit (GPU) and was developed using the PyTorch 2.1.0+cu121 framework. The experimental environment settings are summarized in Table 1.

### 3.3. Evaluation Metrics

To comprehensively evaluate the performance of the proposed model in metal welding defect detection tasks, this paper introduces a series of mainstream quantitative evaluation metrics for object detection, systematically analyzing the model from multiple dimensions, including detection accuracy, model efficiency, and scale adaptability. The metrics used include recall, average precision (AP), mean average precision (mAP), as well as the number of parameters (Params) and computational complexity (GFLOPs) related to actual deployment efficiency [37]. Additionally, to further characterise the model’s detection capabilities under multi-scale targets, multi-scale accuracy metrics are introduced, including mAP50, mAP75, AP_S_, AP_M_, and AP_L_, to refine different aspects of model performance. *Recall* is used to measure the capacity of the model to detect every genuine flaw. It is described as follows:

(5) $$Recall\;=\;\frac{TP}{TP\;+\;FN}$$

*TP* (True Positives) refers to the number of actual defects successfully detected, while *FN* (False Negatives) refers to the number of actual defects that the model failed to identify. A higher Recall indicates a lower false negative rate, demonstrating stronger defect coverage capabilities.

AP (Average Precision) is a core metric that comprehensively evaluates a model’s detection accuracy and recall capability. It is defined as the area under the curve representing the relationship between recall rate and detection performance at different detection thresholds. For refined analysis of accuracy under varying target size conditions, scale-sensitive metrics AP_S_, AP_M_, and AP_L_, which, respectively, measure the model’s detection performance for small-scale (Small), medium-scale (Medium), and large-scale (Large) targets. A higher AP value indicates that the model performs more stably at different confidence levels. The mAP (mean Average Precision) is the most widely used comprehensive evaluation metric in object detection tasks, and it is the average of AP across all categories, defined as follows:

(6) $$mAP\;=\;\frac{1}{N}\sum_{n=1}^{\;N}AP_{n}$$

where *N* is the total number of categories and *AP_n_* is the *AP* value for the nth category. A higher *mAP* indicates better overall detection performance. This paper adopts the official COCO [38] standard to refine this metric. The mAP50 represents the average precision under the condition of IoU ≥ 0.5, reflecting the model’s detection capability under relatively relaxed localization requirements. The mAP75 is the average precision calculated under the condition that the IoU is no less than 0.75, emphasizing the high-precision matching between predicted boxes and ground truth boxes. As a result, it is widely used to evaluate the localization accuracy of a model. The mAP50–95 calculates the average precision at multiple thresholds of IoU ranging from 0.5 to 0.95, enabling a comprehensive assessment of the model’s stable performance across different precision requirements.

### 3.4. Training Parameters and Strategies

All models were trained using a unified hyperparameter configuration to ensure fair comparison. Specific parameters are shown in Table 2.

In the process of choosing hyperparameters, we first referenced the original DEIMs model’s best practices and considered the features of the welding defect object detection task to determine the starting parameters. Then, Hyperparameter tuning was performed on a validation set comprising 20% of the entire dataset, adjusting essential parameters such as the initial learning rate, batch size, and number of training epochs to determine the optimal configuration. For the optimiser selection, the AdamW optimiser [39] was adopted, which is particularly suitable for training deep neural networks in complex object detection tasks. It demonstrates excellent stability and robustness when handling high-noise, high-dimensional feature images of welding defects. In this study, the momentum hyperparameters of AdamW were set to β_1_ = 0.9 and β_2_ = 0.999, which are the commonly adopted default values originating from the Adam optimizer and also widely used in AdamW implementations. These settings have been empirically proven to ensure stable convergence in deep learning tasks and the result indicates that these settings accelerate convergence while maintaining stability in the weight update process. Additionally, AdamW decouples the weight decay term from the gradient update process, effectively enhancing regularisation effects and suppressing overfitting without significantly increasing computational overhead. This feature plays a positive role in addressing common issues in welding defect data, such as small sample sizes, blurred defect boundaries, and noise interference, providing the model with a more robust training foundation.

**Figure 7:**
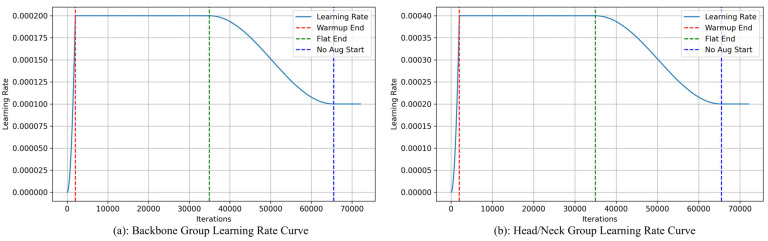
Differential learning rate schedules for different parameter groups. (**a**) Backbone parameters use a lower initial learning rate. (**b**) Other parameters (e.g., head, neck) use a higher initial learning rate.

To achieve optimal model convergence, this experiment adopts a progressive training strategy [40], which achieves optimal results through dynamic parameter adjustments in four stages. During the first four training cycles (0–3 epochs), a linear learning rate warm-up strategy is used, with the base learning rate gradually increasing from 0 to the target value of 4 × 10^−4^. During this stage, all data augmentation operations are disabled, and only fundamental geometric transformations are retained. The sliding average momentum coefficient of the batch normalisation layer is set to 0.9 to stabilise the statistical estimates during the initial phase. This design draws inspiration from He et al.’s [41] initialisation theory to avoid parameter instability caused by gradient fluctuations during early training. After entering the stable training phase (4–63 epochs), the learning rate is kept constant at 4 × 10^−4^, Mixup is activated (α = 0.2), and random erasure and colour jitter are introduced. Upon entering the decay tuning phase (64–119 epochs), the Mixup strength (α) is linearly decayed from 0.2 to 0, the random erasure probability is reduced from 0.2 to 0.05, and the weight decay strength remains unchanged. Cosine annealing learning rate scheduling is initiated, with the formula as follows:

(7) $$\eta_{t}=\;\eta_{0}\cdot \;\frac{1}{2}\left[1\;+\;\cos(\pi \cdot \frac{t-64}{56})\right]$$

where *η*_0_ is the baseline learning rate and *t* represents the current training epoch. This stage was designed according to Loshchilov et al.’s decoupled weight decay theory [39], which maintains regularisation strength while reducing data noise interference during the fine-tuning stage. The final 12 cycles employ a conservative training strategy (epochs 120–131), with the learning rate fixed at 1 × 10^−5^, all data augmentation completely disabled, and weight decay maintained at 1 × 10^−4^. This stage facilitates fine-tuning by eliminating data perturbations, allowing the model to concentrate on key features. Additionally, a grouped learning rate strategy is adopted, with different initial learning rates set for different parameter groups. According to Figure 7, the backbone network is configured with an initial learning rate of 0.0002, while the remaining module parameters use a relatively higher initial learning rate of 0.0004. This strategy effectively prevents the backbone from overfitting during fine-tuning while accelerating the convergence process of newly added modules, thereby further improving the model’s final detection performance while ensuring training stability.

### 3.5. Ablation Experiment

Systematic ablation experiments were conducted to objectively assess the individual contributions and synergistic impacts of the novel modules on model performance. These experiments assessed the effect of each module on the accuracy of object detection, recall capability, and detection speed by sequentially adding or combining each module. The experimental results are shown in Table 3. To verify stability, we repeated the experiments multiple times under identical settings, and the results showed consistently low variations, confirming the robustness of the observed performance gains. First, introducing the SPD-Conv module into the backbone of the base model DEIMs increased the number of parameters from 10.18 M to 10.33 M, while FPS rose slightly by 0.11 and mAP50 improved by 0.2%. Meanwhile, GFLOPs increased from 24.84 to 26.72, but the growth remained moderate compared with the performance gains. These results indicate that SPD-Conv, as a lightweight spatial-aware convolutional structure, enhances object detection accuracy with minimal computational overhead and without significantly increasing model complexity. Second, after adding the EMA attention module, the model parameters and detection speed have, respectively, become 10.39 M and 43.33 FPS, while mAP50 and mAP50–95 improved to 81.7% and 55.4%, respectively, and the recall rate also improved to 79.4%. This confirms that the EMA module effectively enhances the ability to model key features and highlight significant regions. At the same time, GFLOPs increased from 24.84 to 28.83, and FPS decreased compared to the baseline, reflecting the computational cost of feature refinement. Nevertheless, the trade-off between accuracy improvement and complexity remained acceptable. Third, the introduction of the FTPN structure has a particularly significant impact on performance. Compared to the original model, mAP50 improved by 2.5%, mAP50–95 improved by 1.4%, and recall improved to 79.4%. Meanwhile, the number of parameters expanded to 11.52 M, detection speed decreased to 41.57 FPS, and GFLOPs rose to 30.45. These results indicate that FTPN substantially enhances multi-scale feature fusion and detection accuracy, albeit at the cost of increased computational complexity, which accounts for the observed reduction in detection speed.

These results validate the critical role of the improved feature pyramid structure in multi-scale information fusion, improving the model’s response capability when processing different target sizes and enhancing the comprehensiveness of detection coverage. Additionally, when these modules are introduced in paired combinations, model performance further improves without conflicts or performance degradation, indicating their high compatibility and synergistic capabilities. Especially after combining FTPN + EMA, mAP50 and mAP50–95 reach 83.9% and 57.3%, with a recall rate of 79.3%, achieving simultaneous improvements in detection accuracy and recall capability. After incorporating the three improvements, the overall performance of the model was significantly enhanced, with mAP50 and mAP50–95 reaching 84.6% and 58.5%, respectively, and recall improving to 79.9%, representing increases of 3.9%, 3.7%, and 1.4% over the original model. Meanwhile, the parameters increased to 11.87 M, GFLOPs rose to 36.35, and FPS reduced to 35.79 due to the added complexity of EMA and FTPN. Even so, the lightweight design and real-time performance still make the model suitable for deployment on industrial sensors and edge devices, enabling efficient on-site weld defect detection and supporting intelligent sensing applications in manufacturing environments. The overall performance therefore remains within an acceptable range for industrial applications. From the experimental results, the accuracy improvements brought by each module are significant. The final combined model ensures accuracy of detection while simultaneously fulfilling the real-time demands of actual industrial applications, demonstrating a reasonable balance between model efficiency and detection accuracy. Therefore, SPD-Conv, EMA attention mechanism, and FTPN structure each have their advantages in improving model performance. When integrated, good synergistic effects are exhibited, offering a reliable structural foundation for constructing high-accuracy, scalable object detection models.

### 3.6. Comparative Experiment

To evaluate the effectiveness of the proposed enhanced model, DEIM-SFA was comprehensively compared with several advanced object detection models, including Faster R-CNN, RT-DETR-r18, YOLOv8s, the YOLOv10 series (10 s, 10 m, 10 l), and the YOLOv11 series (11 s, 11 m, 11 l). All models were trained on the self-built welding defect dataset under identical hyperparameter settings (input size, optimizer, and data augmentation strategy). The variation was in the number of training epochs: DEIM-SFA and DEIMs were trained for 132 epochs following their official configuration, while the other baselines were trained for 300 epochs. This setup ensured that each model could sufficiently converge and achieve a comparable level of fitting, thereby guaranteeing the fairness of the comparison. The experimental results are shown in Table 4, where DEIM-SFA achieves top results on several essential evaluation criteria, reflecting SOTA performance. Specifically, DEIM-SFA achieves an overall average accuracy of 58.5% on the COCO validation set, ranking first among all comparison models and significantly outperforming mainstream methods such as YOLOv8, YOLOv10, YOLOv11 series, and RT-DETR-r18. For sub-metrics, DEIM-SFA achieves mAP50 and mAP75 values of 84.6% and 63.0%, respectively, representing significant improvements in both object classification and localization accuracy over the competing methods. Regarding scale adaptability, DEIM-SFA achieves detection accuracies of 41.3% and 66.3% for medium-sized targets (AP_M_) and large targets (AP_L_), respectively, both of which are the best among all models. For small object detection, DEIM-SFA achieves an AP_S_ of 24.1%, slightly lower than RT-DETR-r18 but still leading, indicating its strong capability in detecting fine-grained minor defects. In terms of computational resources, DEIM-SFA maintains low model complexity and computational overhead while ensuring high accuracy, with 11.87 million parameters and 36.35 GFLOPS, comparable to the lightweight model YOLOv8s. However, significantly higher accuracy is achieved, demonstrating a good balance between performance and efficiency and possessing excellent potential for engineering deployment. Compared to the baseline model DEIMs, DEIM-SFA improves the mAP50–95 metric by 3.7% across all defect types, with mAP50 improving by 3.9%, mAP75 by 4.3%, and recall by 1.4%. Although the introduction of multiple improvement modules slightly increases the number of parameters and computational complexity, the trade-off is reasonable and entirely acceptable given the performance gains achieved.

**Figure 8:**
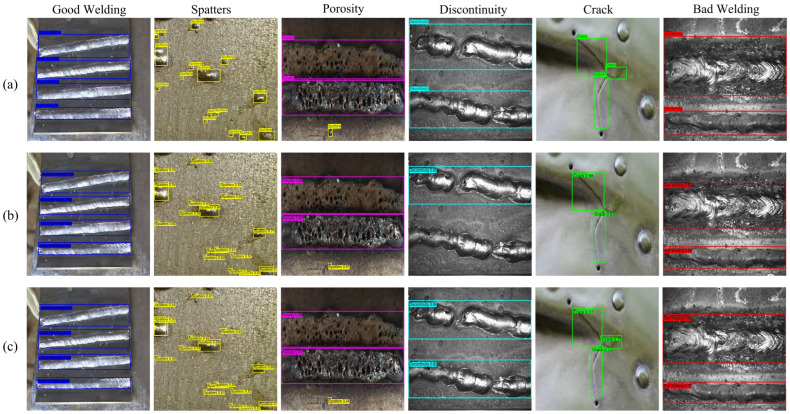
DEIM-SFA and DEIMs’ detection performance comparison. (**a**) True label; (**b**) DEIMs; (**c**) DEIM-SFA.

To visually demonstrate the effectiveness of the improved model in weld defect detection, we compared the detection results of the baseline model and the improved model, using manually annotated ground truth labels as a reference. As shown in Figure 8, through a qualitative analysis of the detection results, the effectiveness of the proposed improvement can be observed. Analysis indicates that the baseline model DEIMs exhibits several shortcomings when handling small-sized and densely distributed defects. It fails to detect small cracks and welding discontinuities, resulting in false negatives. Additionally, in multi-object scenarios, its bounding box regression accuracy is low, frequently leading to class misclassification. In contrast, DEIM-SFA demonstrates exceptional robustness in the same challenging scenarios. The model not only reliably detects small targets that were previously missed but also precisely locates each instance in defect-dense regions, with its predicted bounding boxes highly consistent with the true labels. In terms of improving the reliability of sensing systems, DEIM-SFA significantly reduces false negatives and false positives in complex industrial scenarios, effectively enhancing the measurement credibility and operational stability of vision-based sensing systems, which is of great value in ensuring production safety. These performance improvements can be attributed to the collaborative architecture optimisation that was proposed. The SPD-Conv module preserves fine-grained features essential for small object identification through lossless spatial-to-channel downsampling; the EMA attention mechanism strengthens the network’s emphasis on real defect regions and effectively suppresses background texture interference through spatially aware feature refinement; the FTPN neck network, with its powerful deep fusion capabilities, ensures the model’s precise representation of multi-scale targets in complex scenes. These visualization results strongly validate the superiority of DEIM-SFA in detection accuracy and stability, and intuitively reveal the mechanism of collaborative action among the modules.

## 4. Conclusions

This paper addresses key challenges in industrial welding scenarios, such as low feature recognition accuracy in complex backgrounds and insufficient multi-scale information fusion, by proposing a high-performance detection network named DEIM-SFA. The model achieves this through systematic optimisation of the baseline architecture DEIMs, incorporating three structural innovations: (1) introducing an information-lossless SPD-Conv downsampling module, inspired by the space-to-depth concept, to better preserve the fine-grained features required for identifying minor defects; (2) integrating an improved EMA attention mechanism, derived from existing attention structures but enhanced with spatial perception capability, thereby enabling adaptive refinement of key features in complex weld textures; (3) constructing an FTPN deep fusion neck to achieve more robust and efficient multi-scale feature interaction; (4) systematically combining these modules within DEIM for the first time to construct a principled, task-oriented framework tailored to welding defect detection. Extensive experimental and analytical results demonstrate that the proposed DEIM-SFA significantly outperforms various mainstream detectors, including the latest YOLO series and RT-DETR, across multiple core evaluation metrics. Especially when handling defects with small sizes, blurred boundaries, and complex textures, the model strikes a better balance between computational economy and performance, and it exhibits remarkable detection accuracy.

In practical deployment, DEIM-SFA can serve as an intelligent visual sensing node on edge devices within Industrial Internet of Things systems, providing high-precision defect detection data to enhance product quality, operational safety, and structural lifespan, thereby supporting smart manufacturing and critical infrastructure monitoring. Although this study focuses on visual sensing, the modular framework of DEIM-SFA can be extended to other sensing modalities, offering great potential in multimodal sensing applications. The overall performance of the improved model has been enhanced, but there are still some limitations, especially in terms of detection speed, which still has room for improvement. In future work, a systematic analysis of key parameters will be conducted. Such a sensitivity study is expected to provide valuable insights into the robustness and optimal configuration of DEIM-SFA, further guiding model design and deployment. In addition, lightweight techniques such as model quantization and pruning will be explored to enable real-world deployment and validation, including assessments of memory consumption, latency, and robustness in practical industrial environments, while also integrating multimodal sensor data to enhance model stability and expand its application scope in more complex and diverse industrial scenarios.

## Figures and Tables

**Table 1:** Experimental platform environment configuration.

Component	Specification
Operating System	Ubuntu 22.04 LTS
GPU	NVIDIA RTX 3090
CPU	15 vCPU Intel (R) Xeon (R) Platinum 8358P
Storage	30 GB (System Disk), 50 GB (Data Disk)
RAM	90 GB
Framework	PyTorch 2.1.0
CUDA	12.1
Python Version	3.10.8

**Table 2:** Experimental parameters.

Parameter	Value	Parameter	Value
Epoch	132	Betas	(0.9, 0.999)
Batch size	8	Weight decay	1 × 10^−4^
Optimizer	AdamW	Warmup epoch	3
Initial learning rate	4 × 10^−4^	Image size	640 × 640

**Table 3:** The ablation experiment results.

Methods	mAP50	mAP50–95	Recall	Params/M	FPS	GFLOPS
DEIMs	80.7	54.8	78.4	10.18	51.18	24.84
DEIMs + A	80.9	54.1	78.5	10.33	51.29	26.72
DEIMs + B	81.7	55.4	79.4	10.39	43.33	28.83
DEIMs + C	83.2	56.2	79.4	11.52	41.57	30.45
DEIMs + A + B	81.9	55.3	79.7	10.54	49.27	30.71
DEIMs + A + C	82.1	56.0	78.9	11.66	40.07	32.36
DEIMs + B + C	83.9	57.3	79.3	11.73	35.81	34.45
DEIMs + A + B + C	84.6 (+3.9)	58.5 (+3.7)	79.8 (+1.4)	11.87	35.79	36.35

Note: A represents the SPD-Conv module, B represents the EMA attention module, and C represents the FTPN structure.

**Table 4:** Comparison of several model algorithms.

Model	Params	GFLOPS	mAP_50–95_	mAP_50_	mAP_75_	AP_S_	AP_M_	AP_L_
Faster-RCNN	137.1	370.2	46.7	74.6	44.3	14.7	31.9	54.8
RT-DETR-r18	19.89	57.0	55.3	81.1	58.8	**24.5**	40.9	65.7
YOLOv8s	11.13	28.4	55.6	82.5	60.0	20.2	38.2	64.1
YOLOv10s	**8.07**	24.8	52.6	79.7	56.9	22.4	34.7	61.2
YOLOv10m	16.49	64.1	54.5	80.9	59.1	23.0	40.9	58.0
YOLOv10l	25.77	127.2	53.9	80.1	58.8	22.8	37.8	60.9
YOLOv11s	9.43	**21.5**	48.7	74.8	50.7	22.9	32.1	55.7
YOLOv11m	20.03	67.7	46.6	77.1	46.4	20.0	32.3	48.2
YOLOv11l	25.28	86.6	53.0	80.3	56.4	20.2	33.0	58.9
DEIMs	10.18	24.84	54.8	80.7	58.7	23.5	39.8	63.9
DEIM-SFA	11.87	36.35	**58.5**	**84.6**	**63.0**	24.1	**41.3**	**66.3**

Note: Bold data is the best value of each indicator.

## Data Availability

The data that support the findings of this study are available from the corresponding author, Y.G., upon reasonable request.

## Abbreviations

The following abbreviations are used in this manuscript:

SPD-Conv	Spatial-depth conversion convolution
EMA	Efficient multi-scale attention
CNN	Convolutional neural network
AP	Average Precision
mAP	Mean Average Precision
COCO	Common Objects in Context
FPN	Feature Pyramid Network
PANet	Path Aggregation Network
FPS	Frames Per Second
GFLOPs	Giga Floating Point Operations
IoU	Intersection over Union
ESE	Efficient Squeeze-and-Excitation
GPU	Graphics Processing Unit
SSD	Single-Shot MultiBox Detector
